# Functional Changes of the Community of Microbes With Ni-Dependent Enzyme Genes Accompany Adaptation of the Ruminal Microbiome to Urea-Supplemented Diets

**DOI:** 10.3389/fmicb.2020.596681

**Published:** 2020-12-22

**Authors:** Zhongyan Lu, Zhihui Xu, Lingmeng Kong, Hong Shen, Jörg R. Aschenbach

**Affiliations:** ^1^Key Laboratory of Animal Physiology and Biochemistry, College of Veterinary Medicine, Nanjing Agricultural University, Nanjing, China; ^2^College of Life Sciences, Nanjing Agricultural University, Nanjing, China; ^3^Bioinformatics Center, Nanjing Agricultural University, Nanjing, China; ^4^Institute of Veterinary Physiology, Freie Universität Berlin, Berlin, Germany

**Keywords:** ruminal microbiome, urea supplementation, Ni-dependent enzyme, rumen, functional changes

## Abstract

Urea is an inexpensive non-protein nitrogen source commonly supplemented to the diets of ruminants. It is cleaved to ammonia by bacterial ureases, which require Ni as a catalyst for ureolysis. The key event in the changes of the ruminal microbiome after urea supplementation remains unknown. We have therefore investigated changes in the ruminal microbiome and its community with Ni-dependent enzyme genes following urea supplementation and analyzed the associations of rumen environmental factors, including fermentation variables and Ni concentrations, with the compositional and functional changes of these communities. We found that urea supplementation increased urease activity and the concentrations of ammonia and Ni, and tended to increase concentrations of short chain fatty acids and acetate, whereas it decreased rumen pH and the L-/D-lactate ratio. With standards for genome completeness >60% and strain heterogeneity <10%, 20 bacterial species containing five Ni-dependent enzyme genes were detected in the metagenome sequences. For the five Ni-dependent enzyme genes, urea supplementation increased the relative abundances of genes of urease and acetyl-CoA synthase, whereas it decreased the relative abundances of genes of glyoxalase I, [NiFe]-hydrogenase, and lactate racemase. For the 20 microbes with Ni-dependent enzyme genes, urea supplementation increased the relative abundances of five bacteria exhibiting high capacities for the utilization of hemicellulose and pectin for butyrate and fatty acid biosynthesis. For the ruminal microbiome, urea supplementation increased the metagenomic capacities for hemicellulose and pectin degradation, butyrate generation, fatty acid biosynthesis, and carbon fixation, whereas it decreased the metagenomic capacities for starch degradation, propionate generation, and sulfur and nitrogen metabolism. Constrained correspondence analysis identified rumen ammonia and Ni concentrations as likely driving factors in the reshaping of the ruminal microbiome and, together with pH, of the community of microbes with Ni-dependent enzyme genes. Thus, the functional change of the latter community is probably an important event in the adaptation of the ruminal microbiome to urea-supplemented diets. This result provides a new perspective for the understanding of the effects of urea supplementation on rumen fermentation.

## Introduction

Ruminants carry a characteristic microbiome in their rumen which is primarily influenced by ruminant species and diet. Diet effects are most prominent on bacterial communities, which probably reflects the very diverse metabolic capabilities of bacteria as compared to archaea and protozoa (Henderson et al., 2016). Ureolysis is one of the key metabolic feature of a small group of rumen bacteria, occupying less than 0.1% of the total count of rumen microbes. However, ureolysis has functional implications for nitrogen recycling and microbial protein synthesis with expectable effects on a wide range of other metabolic processes in the rumen (Patra and Aschenbach, 2018).

The usability of urea for *de novo* synthesis of microbial protein makes urea an inexpensive non-protein nitrogen source that is commonly supplemented to the diets of ruminants to improve the ruminal nitrogen balance. By substituting true proteins (TP) in the diet, urea supplementation concurrently reduces feed costs. Studies have shown that urea supplementation alters the diversity and structure of the ruminal microbiome but has almost no impacts on the abundance of ureolytic bacteria when compared with animals fed diets without urea supplementation (Jin et al., 2017). Thus, the question as to how the dietary supplementation of urea reshapes the structure of the ruminal microbiome without impacting on the composition and abundance of ureolytic bacteria remains open.

Urease, which hydrolyzes urea into ammonia and bicarbonate, is a nickel (Ni)-dependent enzyme that requires the metal cofactor Ni in the catalytic center for biochemical reaction (Ragsdale, 2007; Boer et al., 2014). An earlier study found that ruminal urease activity was extremely low in lambs receiving a diet with low Ni content, but was many times higher in lambs receiving a diet with Ni supplementation (Spears et al., 1977). *In vitro* studies further identified that bacterial urease activity was positively regulated by the availability of Ni as cofactor via the NikR regulatory protein, and reduced urease activities suppressed the growth of ureolytic bacteria in the GI tract due to acid sensitivity (Nolan et al., 2002; Ernst et al., 2005). Sequencing of the ureC amplicon revealed that bacteria with urease genes were abundant and diverse in the rumen (Jin et al., 2017), whereas the ruminal urease activity is known to be low in basal diets without Ni supplementation (Spears and Hatfield, 1978). Thus, the availability of Ni is probably an important factor affecting not only the urease activity but also the population size of individual ureolytic bacteria in the rumen. Urease is not the only Ni-dependent enzyme produced by bacteria and archaea. So far, eight other Ni-dependent enzymes have been identified, including (1) lactate racemase, which catalyzes the conversion between L-lactate and D-lactate; (2) carbon monoxide dehydrogenase, which catalyzes the interconversion of carbon monoxide (CO) and carbon dioxide (CO_2_); (3) acetyl-CoA synthase, which catalyzes the synthesis of acetyl-CoA from CO_2_; (4) glyoxalase I, which catalyzes the conversion of hemithioacetal to 2-hydroxythioester; (5) Ni acireductone dioxygenase, which catalyzes the conversion of acireductone to 3-(methylthio)propionate, formate, and CO; (6) methyl-CoM reductase, which catalyzes the interconversion of methyl-CoM plus CoB with methane and heterodisulfide CoM-S-S-CoB; (7) Ni superoxide dismutase, which catalyzes the conversion of superoxide to oxygen (O_2_) and hydrogen peroxide (H_2_O_2_); and (8) [NiFe]-hydrogenase, which catalyzes the production and consumption of hydrogen gas. Although there is no report concerning the kinds and roles of the microbes with these Ni-dependent enzyme genes in the rumen, these enzymes catalyze vital reactions in energy and substrate metabolism and are crucial for the function and survival of the microbes (Ragsdale, 2007; Maroney and Ciurli, 2014). Thus, acquisition of Ni should also be important for the colonization of microbes with these Ni-dependent enzyme genes in the rumen.

The availability of Ni is important for the growth of microbes with Ni-dependent enzyme genes. However, Ni is neither secreted nor stored by the mammalian host (Sunderman, 1993). Thus the prime source of rumen Ni is from dietary intake. Urea supplementation was shown to improve the feed intake and nutrients digestibility of ruminants receiving sufficient energy source (Zhou et al., 2017), which might affect the availability of Ni in the rumen. In addition, urea-supplemented diets are usually designed to be isonitrogenous or isoenergetic with the control diet, bringing about differences in the composition of dietary ingredients with possible impact on dietary Ni content (Zhou et al., 2017; Xu et al., 2019). Accordingly, we hypothesized that urea supplementation might affect ureolytic activity and the composition of the microbial community with genes encoding for urease and other Ni-dependent enzymes and that such changes may be relevant for the adaptation of the whole ruminal microbiota. To test this hypothesis, we investigated the kinds of microbes with the Ni-dependent enzyme genes using metagenome sequencing and compared the functions of the community consisting of these microbes with that of the ruminal microbiome. We further analyzed the associations with rumen environmental variables, including rumen fermentation indices and Ni concentration, in the shaping of these two communities in goats fed isonitrogenous diets without or with 0.5% urea supplementation. Our aim was to generate a better understanding of the effects of urea supplementation on rumen fermentation.

## Materials and Methods

### Ethics Approval

All experimental procedures involving animals were approved by the Animal Care and Use Committee of Nanjing Agricultural University (SKSH2019-0104), in compliance with the Regulations for the Care and Use of Animals (Nanjing Agricultural University, 1999) and the Regulations for the Administration of Affairs Concerning Experimental Animals (NO. 2 Document of the State Science and Technology Commission of China, 1998).

### Animals and Diets

Twelve goats (Boer × Yangtze River Delta White, 4 months, 14–16 kg bodyweight) were obtained from the Liuhe Goat Farm (Nanjing, Jiangsu, China). They were maintained in individual boxes with free access to water in the animal house of Nanjing Agricultural University. After a 14-day adaptation to a hay diet, goats were randomly allocated into two groups receiving a diet containing either 0% urea (true protein; TP group, *n* = 6) or 0.5% urea (urea supplementation; US group, *n* = 6). To effectively compare the effects of the two N sources, rather than the increase of total N content, on the rumen fermentation, the diets were designed to be isonitrogenous (containing the same N content but different true protein content) and met nutrient requirements in compliance with the feeding standard of meat-producing goats (NY/T816-2004). The ingredients and chemical compositions of the two diets are listed in Table 1.

**TABLE 1:** Feed intake of goats and composition of experimental diets.

Item	TP	US
DM Intake, g/day	787	859
**Ingredient, % of DM**		
Grass hay (*Leymus chinensis*)	56.6	57.9
Corn meal	24.1	24.6
Soya bean meal	10.7	8.2
Wheat bran	6.7	6.8
Urea^1^	0.0	0.5
Mineral and vitamin supplement^2^	1.0	1.0
Salt	0.3	0.3
CaHPO_4_	0.8	0.8
**Chemical composition**		
DM,%	89.0	88.8
Crude protein, %DM	12.4	12.6
Crude fat, %DM	3.1	3.0
Ash, %DM	6.1	5.1
NDF, %DM	48.8	48.9
ADF, %DM	21.0	21.2
NFC^3^, %DM	34.4	31.3
Ni, mg/kg DM	2.25	2.32

*DM, dry matter; NDF, neutral detergent fiber (analyzed with amylase, inclusive of residual ash); ADF, acid detergent fiber; NFC, non-fiber carbohydrate.^1^Urea concentration is provided as 5.0 g/kg DM.^2^Contained 16% calcium carbonate, 102 g/kg Zn, 47 g/kg Mn, 26 g/kg Cu, 1,140 mg/kg I, 500 mg/kg Se, 340 mg/kg Co, 17,167,380 IU/kg vitamin A, 858,370 IU/kg vitamin D, and 23,605 IU/kg vitamin E.^3^NFC = 100 – (NDF + crude protein + crude fat + ash).*

To avoid selection of dietary components, a total mixed ration (TMR) was offered at 0700 and 1900 daily during the experiment. Feed intake and refusal by individual goats were measured daily during the experiment. The amount of feed offered during the experimental period was adjusted on a weekly basis to allow for ∼10% orts (uneaten portion of the daily feed ration). Feeds were sampled at the beginning and end of the experiment. Dry matter, ash, crude fat, and crude protein contents of samples were analyzed according to the procedures of AOAC (Cunniff and International, 1997). Acid detergent fiber (ADF) and neutral detergent fiber (NDF) values were analyzed according to the procedures of Van Soest et al. (1991). Dietary Ni concentration was determined using an atomic absorption spectrometry (PE Model 5000, PerkinElmer Inc., Shelton, CT, United States) fitted with a nickel hollow cathode lamp according to the method described by Rahnama and Najafi (2016).

### Sample Collection

All goats were killed at the local slaughterhouse at 6 h after receiving their morning feed on day 28. Rumen contents were strained through a four-layer cheesecloth filter and immediately subjected to pH measurement. Four aliquots (10 mL) of rumen fluid were stored at −20°C for the extraction of microbial DNA, the determination of short-chain fatty acids (SCFA) concentrations, the determination of urease activity, ammonia and lactate concentrations, and the determination of Ni concentration.

### Microbial DNA Extraction and Metagenome Shotgun Sequencing

We used the bead-beating method to break the cell walls and release nucleic acid material of microbes according to a protocol modified from Zoetendal et al. (1998). In brief, 10 ml of fluid samples were centrifuged at 900 × *g* for 5 min. The pellets were resuspended in 1 ml of the stabilizer buffer from Bacterial DNA Kit (Omega, Shanghai, China). After the addition of 0.3 g of metal beads (diameter 0.1 mm), samples were beaten for 3 min in a Mini-beadbeater-16 (BioSpec, Bartlesville, OK, United States), and centrifuged for 10 min at 18,000 × *g*. The supernatant was then used for DNA isolation following the instruction of Bacterial DNA Kit (Omega, Shanghai, China). The DNA concentration was determined in a NanoDrop 1000 (Thermo Fisher Scientific, Wilmington, DE, United States). The integrity of the microbial DNA was evaluated on a 1.0% agarose gel. DNA libraries were constructed by using the TruSeq DNA Sample Prep Kit (Illumina, San Diego, CA, United States) and then sequenced via paired-end chemistry (PE150) on the Illumina HiSeq X platform (Illumina, San Diego, CA, United States) at Biomarker Technologies (Beijing, China).

### Analyses of Urease Activity, Concentrations of Ni, Lactate, Ammonia, and SCFA, and Rumen pH

Rumen SCFA concentrations were determined using a gas chromatograph (HP6890N, Agilent Technologies, Wilmington, DE, United States) as described by Yang et al. (2012). Rumen Ni concentration was determined in the supernatant of a 10 ml rumen fluid sample (centrifugation at 900 × *g* for 5 min) with the atomic absorption spectrometry fitted with a nickel hollow cathode lamp according to the method described by Rahnama and Najafi (2016). Concentrations of L-lactate, D-lactate, and ammonia, and urease activity were determined by using the L-Lactate Assay Kit, D-Lactate Assay Kit, Ammonia Assay Kit, and Urease Activity Assay Kit (ab65331, ab83429, ab83360, and ab204697, Abcam Trading Co., Ltd., Shanghai, China), respectively, and a microplate reader (Infinite M200 PRO, Tecan Trading AG, Switzerland). SPSS V21.0 (SPSS Inc., Chicago, IL, United States) was applied for statistical comparison using the two-tailed *t*-test. Differences were considered significant when *P* < 0.05 in the two-tailed *t*-test.

### Metagenomic Data Analysis

#### Genome Assembly

Raw reads were filtered using FastX v0.0.13 (Gordon and Hannon, 2010), with a quality cutoff of 20. Reads shorter than 30 bp were discarded from the sample. The reads that were considered to have originated from the host and feeds were removed by using DeconSeq v0.4.3 (Schmieder and Edwards, 2011), with the NCBI goat, corn, soybean, and grass genome sequences as references. The remaining high-quality reads of all samples were then assembled into scaffolds by using IDBA-UD 1.1.1 (Peng et al., 2012).

#### Non-redundant (NR) Gene Set Construction

Genes were predicted from the scaffolds by using FragGeneScan 1.31 (Rho et al., 2010). Predicted genes from all samples were gathered together to form a large gene set. CD-HIT (Fu et al., 2014) was used to construct the NR gene set by setting 90% identity and 90% coverage of the gene with the longer sequences in the clustering.

#### Gene Abundance Calculation

High-quality reads of each sample were mapped to the NR gene set by using Bowtie2 v2.3.4 (Langmead and Salzberg, 2012). MarkDuplicates in the Picard toolkits version 2.0.1^1^ was used to remove the PCR duplicates in the reads, and HTSeq v0.9.1 (Anders et al., 2015) was then employed to calculate the gene count. The transcripts per kilobase of exon model per million mapped reads (TPM) of the gene, calculated as [(gene count/gene length) × 10^6^/sum (gene count/gene length)], were used to normalize the gene abundance between the genes and treatments. The abundance of genes was compared by using R program DESeq2 package (Love et al., 2014). Differences were considered significant when false discovery rate (FDR) was <0.05.

#### Gene Function Annotation

The Kyoto Encyclopedia of Genes and Genomes (KEGG) Orthologies (KO) terms of predicted genes were obtained by using the KEGG Automatic Annotation Server (KAAS) (Moriya et al., 2007). Subsequently, KO terms were mapped to KEGG pathways via the KEGG mapper provided by the KEGG website.

#### Taxonomic Annotation and the Abundance Calculation of Microbes With Ni-Dependent Enzyme Genes

The high-quality reads of each sample were mapped back to the scaffolds by using Bowtie2. The calculation of TPMs of scaffolds was consistent with that of the genes. The abundance of scaffolds was compared by using DESeq2 package. Differences were considered significant when FDR < 0.05. The composition-based rank-flexible classifier Epsilon-NB in FCP v1.0.7 (Parks et al., 2011) was used to assign the taxonomy of the scaffolds, with the in-house reference catalog of 12,607 bacterial and 21,537 archaeal complete genomes downloaded from the NCBI RefSeq database^2^. The Ni-dependent enzyme genes in the NR gene set were found according to the KO numbers. Next, the available sequences of Ni-dependent enzyme genes were downloaded from the UniProt dataset^3^ and blasted with the corresponding gene sequences in the NR gene set. The matched genes, with identity >85% and coverage >90%, were retrieved and aligned in MUSCLE version 3.8.31 (Edgar, 2004). The maximum likelihood trees of each investigated gene set were constructed by using the LG model in PhyML 3.0 (Guindon et al., 2010).

#### Functional Comparison of Microbes With Ni-Dependent Enzyme Genes

CONCOCT 1.0.0 (Alneberg et al., 2014) was used for the binning of scaffolds. CheckM (Parks et al., 2015) was used to estimate the completeness, contamination, and strain heterogeneity of the obtained bins. Bins with >60% completeness, <10% contamination, and <10% strain heterogeneity were used in the following analysis. The bins annotated as the same species were summed. The KO terms contained in the sequences of individual species were mapped to a self-constructed SCFA generation pathway and to KEGG sulfur metabolism, nitrogen metabolism, and FA biosynthesis pathways. The pathway completeness was calculated as the number of pathway enzyme genes/average completeness of bins. Finally, the analysis of variance (ANOVA) in SPSS V21.0 was applied to compare the metabolic abilities among the populations consisting of microbes with Ni-dependent enzyme genes. The differences were considered significant when FDR < 0.05.

#### Comparisons of the Structure and Diversity of the Ruminal Microbiome and the Community With Ni-Dependent Enzyme Genes

The non-metric multidimensional scaling (NMDS) plot obtained by using the R vegan package (Oksanen et al., 2016) was employed to compare the structures of the ruminal microbiome and of the community of microbes with Ni-dependent enzyme genes between treatments. Shannon and Simpson indices, calculated by using the R program phyloseq package (McMurdie and Holmes, 2013), were used to compare the diversity of the communities between treatments. Differences were considered significant when *p* < 0.05 in the two-tailed *t*-test.

#### KEGG Pathway Enrichment Analysis

The number of significantly increased/decreased genes, the number of occurring genes in a specific metabolism pathway, and the number of genes in the pathway were gathered to analyze the upregulation/downregulation of the specific pathway between groups by using the hypergeometric test. Differences were considered significant when FDR < 0.05.

#### Effects of Environmental Factors on the Structure of the Ruminal Microbiome and the Community of Microbes With Ni-Dependent Enzyme Genes

Envfit and constrained correspondence analysis (CCA) functions in the R program vegan package were used to investigate the associations of rumen pH and of ammonia, Ni, D-lactate, L-lactate, and individual SCFA concentrations with the structure of the ruminal microbiome and the community of microbes with Ni-dependent enzyme genes. Associations with environmental factors were considered significant when FDR < 0.05.

## Results

### Effects of Urea Supplementation on the Rumen Environment and Urease Activity

Compared with the values of the TP group, the rumen ammonia concentration, Ni concentration, and urease activity were significantly increased, whereas the rumen pH and the ratio of L-lactate to D-lactate were significantly decreased in the US group (Table 2). The concentrations of acetate, total SCFA, and D-lactate tended to increase, whereas the concentration of L-lactate tended to decrease in the US group. Concentrations of propionate and butyrate showed no significant changes between groups (Table 2).

**TABLE 2:** Comparison of ruminal pH, urease activity and concentrations of ammonia, Ni, lactate and SCFAs between groups.

Item	TP^1^	US^1^	*p*^2^
pH	6.76 ± 0.02	6.67 ± 0.03	0.03
Ammonia, mM	12.65 ± 0.45	16.97 ± 0.90	0.02
Urease activity, U/mL^3^	1.07 ± 0.05	1.31 ± 0.07	0.02
Ni, μM	8.52 ± 0.68	11.91 ± 0.54	0.00
Acetate, mM	51.57 ± 1.02	54.65 ± 1.28	0.08
Propionate, mM	18.83 ± 0.75	20.45 ± 1.53	0.39
Butyrate, mM	11.93 ± 0.43	12.45 ± 0.63	0.60
Total SCFA^4^, mM	82.33 ± 1.49	87.55 ± 2.58	0.10
L-Lactate, mM	0.61 ± 0.04	0.57 ± 0.04	0.07
D-Lactate, mM	0.40 ± 0.02	0.49 ± 0.04	0.08
L-Lactate/D-lactate	1.58 ± 0.15	1.19 ± 0.13	0.04

*^1^Values are mean ± standard error (SE).^2^*P* values were calculated by two-tailed *t*-test.^3^One unit was defined as one μmol ammonia released per min.^4^Total SCFA = acetate + propionate + butyrate.*

### Effects of Urea Supplementation on the Structure and Function of the Ruminal Microbiome

In total, 179 gigabases (GB) high-quality data were obtained from the metagenomic shotgun sequencing; 31 GB data, which were likely to originate from the host and from the composition of the diet, were excluded from the datasets. We assembled 2,238,467 scaffolds from the clean data. In addition to 388 unclassified species, 107 archaeal species belonging to 65 genera and 934 bacterial species belonging to 561 genera were detected in the sequences. Euryarchaeota was the most abundant phylum, and *Thermococcus* was the most abundant genus within the Archaea. For the annotated species, the relative abundances of 63 archaea and 467 bacteria were significantly increased, whereas the relative abundances of 17 archaea and 232 bacteria were significantly decreased in the US group compared with the TP group (Supplementary Table 1). As indicated by Shannon and Simpson indices, the diversity of ruminal microbiome was significantly increased in the US group when compared with that of the TP group (Supplementary Table 2). The NMDS analysis indicated a significant difference of the structure of the ruminal microbiome between treatments (Supplementary Figure 1).

**FIGURE 1:**
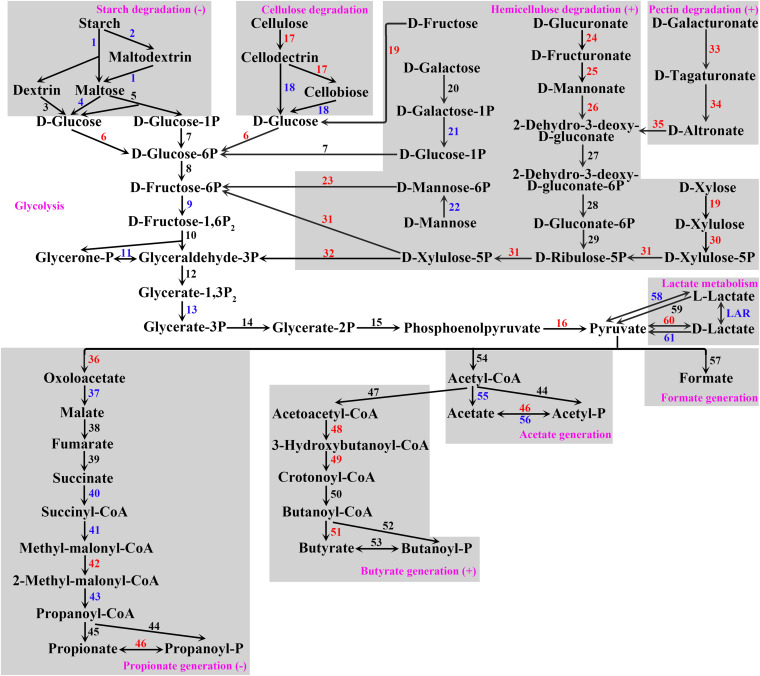
SCFA generation pathway constructed for the ruminal microbiome in the present study. Gray boxes frame sub-pathways, with (–) indicating downregulation and (+) indicating upregulation of sub-pathways by urea supplementation. Pathway enzyme genes are coded by numbers (for code, see Supplementary Table 4) with red indicating increased (FDR < 0.05) and blue indicating decreased (FDR < 0.05) relative abundance of this enzyme gene by urea supplementation. Enzyme genes 27–32 are common enzyme genes of pectin and hemicellulose degradation. Enzyme genes 44 and 46 are common enzyme genes of acetate and propionate generation. For further details of pathway enzyme genes, see Supplementary Table 4.

At the gene level, 2,273,291 open reading frames (ORF) in total were detected in the data, which were annotated to 4,939 KOs. Compared with the TP group, the relative abundances of 1,015 KO were significantly upregulated, and the relative abundances of 1,061 KO were significantly downregulated in the US group (Supplementary Table 3). Since acetate, butyrate, and propionate were the major fermentation products in the rumen, we reconstructed the SCFA generation pathway consisting of 10 sub-pathways according to the KO mapping results and previous studies (McDonald and Warner, 1975; Figure 1). As a result, 62 pathway enzyme genes (PEG) consisting of 101 KO were detected in the SCFA generation pathway (Supplementary Table 4). The relative abundances of 21 PEG were significantly increased, whereas the relative abundances of 17 PEG were significantly decreased in the US group compared with the TP group (Figure 1 and Supplementary Table 4). The hypergeometric tests showed that, among the sub-pathways, hemicellulose degradation, pectin degradation, and butyrate generation were significantly upregulated, whereas starch degradation and propionate generation were significantly downregulated in the US group when compared with the TP group. Among the KEGG amino acid, energy, lipid, nucleotide, and glycan metabolism pathways, eight pathways were significantly upregulated, whereas five pathways were significantly downregulated in the US group when compared with the TP group (Supplementary Figure 2).

### Effects of Urea Supplementation on the Relative Abundance of Ni-Dependent Enzyme Genes

In total, five Ni-dependent enzyme genes were detected in the data with the most abundant representation being those of genes encoding for [NiFe]-hydrogenase (*HYD*) and glyoxalase I (*GLO*) (Table 3). Sequences of genes encoding for Ni acireductone dioxygenase (*Ni-ARD*), methyl-CoM reductase (*MCR*), and Ni superoxide dismutase (*Ni-SOD*) were not detected in our data. Sequences of genes encoding for urease (*URE*) and bifunctional acetyl-CoA synthase*/*carbon monoxide dehydrogenase (hereinafter referred to as *ACS*) were significantly increased in the US group compared with the TP group based on the average abundance of detectable enzyme subunit genes plus their accessory protein subunit genes and maturation factor subunit genes. On the contrary, the average abundance of detectable *HYD* subunit genes, the relative abundance of *GLO* and the relative abundance of the gene encoding for lactate racemase (*LAR*) were significantly decreased in the US group compared with the TP group.

**TABLE 3:** Comparison of the relative abundance of detectable subunit genes plus biosynthesis and maturation-related protein genes of the nickel-dependent enzymes as well as the average abundance of such gene groups.

			Subunit		Average	
			
	KO	Gene	TP (TPM)	US (TPM)	TP (TPM)	US (TPM)
Urease	K01428	urease subunit alpha gene (*ureC*)	0.30 ± 0.09	1.13 ± 0.20^1^	0.18 ± 0.02	0.55 ± 0.081
	K01429	urease subunit beta gene (*ureB*)	0.17 ± 0.04	0.91 ± 0.19^1^		
	K01430	urease subunit gamma gene (*ureA*)	0.26 ± 0.06	0.63 ± 0.09^1^		
	K03188	urease accessory protein gene (*ureF*)	0.02 ± 0.01	0.28 ± 0.05^1^		
	K03189	urease accessory protein gene (*ureG*)	0.25 ± 0.08	0.26 ± 0.05		
	K03190	urease accessory protein gene (*ureD/ureH*)	0.08 ± 0.02	0.19 ± 0.04^1^		
Acetyl-CoA synthase/CO-dehydrogenase	K00192	anaerobic carbon-monoxide dehydrogenase subunit alpha gene (*cdhA*)	0.06 ± 0.01	0.09 ± 0.00	2.11 ± 0.14	5.04 ± 0.11^1^
	K00198	anaerobic carbon-monoxide dehydrogenase catalytic subunit gene (*acsA*)	6.13 ± 0.39	14.78 ± 0.28^1^		
	K00196	anaerobic carbon-monoxide dehydrogenase iron sulfur subunit gene (*cooF*)	0.16 ± 0.02	0.22 ± 0.07^1^		
[NiFe]-Hydrogenase	K04651	hydrogenase nickel incorporation protein gene (*HypA*)	4.50 ± 0.43	1.21 ± 0.27^1^	56.73 ± 3.52	32.68 ± 0.74^1^
	K04652	hydrogenase nickel incorporation protein gene (*HypB*)	135.65 ± 6.38	158.32 ± 4.15^1^		
	K04653	hydrogenase expression/formation protein gene (*HypC*)	24.47 ± 2.71	2.33 ± 0.40^1^		
	K04654	hydrogenase expression/formation protein gene (*HypD*)	60.66 ± 7.13	10.40 ± 0.65^1^		
	K04655	hydrogenase expression/formation protein gene (*HypE*)	60.07 ± 5.95	7.36 ± 0.49^1^		
	K04656	hydrogenase maturation protein gene (*HypF*)	55.03 ± 5.87	12.27 ± 0.42^1^		
Glyoxalase I	K01759	lactoylglutathione lyase gene (*gloA*)	104.22 ± 8.53	36.57 ± 1.47^1^		
Lactate racemase	K22373	lactate racemase gene (*larA*)	19.61 ± 1.77	11.76 ± 0.78^1^		

*Values are mean ± standard error (SE).^1^*FDR* < 0.05.*

### Effects of Urea Supplementation on the Composition of the Community of Microbes With Ni-Dependent Enzyme Genes

In order to elucidate the composition of the community of microbes with Ni-dependent enzyme genes, binning was performed to compute the scaffolds that likely originated from the same species, followed by the phylogenetic analysis of Ni-dependent enzyme genes to further our understanding of the taxonomy of species. In total, 57 clusters (bins) that had more than 60% completeness, less than 10% contamination, and less than 10% strain heterogeneity were found to contain scaffolds with Ni-dependent enzyme genes (Supplementary Table 5). These 57 bins covered 22.6–28.8% of all *URE*, 41.3–47.1% of all *ACS*, 38.2–49.4% of all *GLO*, 3.5–13.6% of all *HYD*, and 18.8–21.2% of all *LAR* abundance detected in the metagenome sequences. The results of the maximum likelihood (ML) analysis of the Ni-dependent enzyme genes detected in this study with that downloaded from the UniProt dataset supported the taxonomic classification of the clusters obtained in previous steps.

According to the binning results, the Ni-dependent microbial community consisted of 57 bins belonging to 20 species (Table 4). Among them, the relative abundances of five species (referred to as the upregulated population) were significantly increased, whereas the relative abundances of nine species (referred to as the downregulated population) were significantly decreased in the US group compared with the TP group (Table 4). The relative abundances of the remaining six species showed no significant difference between groups (referred to as the unchanged population). Furthermore, the composition of the community of microbes with Ni-dependent enzyme genes differed between treatments; however, the diversity indices (Shannon and Simpson) were not significantly different between treatments (Supplementary Figure 1 and Supplementary Table 2).

**TABLE 4:** Comparison of the relative abundances and the compositions of Ni-dependent enzyme genes of the 20 Ni-dependent bacteria.

Population	Species	Ni-dependent enzyme genes	Relative abundance		*FDR*
			TP (TPM)	US (TPM)	
Upregulated	Clostridiales^1^	*ACS*	12.24 ± 1.01	48.36 ± 2.02	0.00
	*Prevotella*^1^	*GLO*	4.13 ± 0.54	22.17 ± 0.72	0.00
	*Clostridium*^1^	*ACS, HYD, GLO, LAR*	9.28 ± 1.22	20.98 ± 0.79	0.03
	*Ruminococcus*^1^	*ACS, HYD, GLO*	0.32 ± 0.01	9.94 ± 0.64	0.00
	*Butyrivibrio*^1^	*HYD, GLO, LAR*	0.93 ± 0.06	8.00 ± 0.35	0.00
Downregulated	*Prevotella ruminicola*^2^	*GLO*	96.46 ± 3.64	27.63 ± 0.76	0.00
	*Selenomonas ruminantium*^2^	*HYD, GLO, LAR*	47.07 ± 5.26	5.76 ± 0.22	0.02
	*Caloramator*^2^	*HYD, LAR*	23.86 ± 2.95	0.04 ± 0.00	0.02
	*Selenomonas sputigena*^2^	*HYD, GLO, LAR*	10.13 ± 1.33	1.66 ± 0.07	0.04
	*Succiniclasticum ruminis*^2^	*HYD, GLO, LAR*	6.97 ± 0.78	1.37 ± 0.10	0.03
	*Ruminococcus albus*^2^	*HYD, GLO*	4.72 ± 0.28	0.81 ± 0.05	0.00
	*Treponema*^2^	*GLO*	2.72 ± 0.18	0.95 ± 0.08	0.01
	*Fibrobacter succinogenes*^2^	*GLO*	1.77 ± 0.07	0.14 ± 0.01	0.00
	*Desulfitobacterium hafniense*^2^	*GLO, LAR*	1.55 ± 0.08	0.36 ± 0.02	0.00
Unchanged	*Clostridium proteoclasticum*	*HYD, GLO*	12.97 ± 4.11	0.11 ± 0.02	0.92
	*Lachnoclostridium*	*LAR*	10.80 ± 1.49	5.67 ± 0.37	0.58
	*Clostridium thermocellum*	*HYD*	2.87 ± 0.30	1.66 ± 0.17	0.83
	*Clostridium beijerinckii*	*LAR*	2.69 ± 0.53	0.08 ± 0.02	0.17
	*Treponema succinifaciens*	*URE*	1.31 ± 0.10	3.05 ± 0.22	0.07
	*Sphaerochaeta globus*	*LAR*	0.67 ± 0.10	0.24 ± 0.01	0.28

*^1^Relative abundance increased by urea supplementation; ^2^Relative abundance decreased by urea supplementation.*

To obtain a general idea concerning the metabolic capabilities of these Ni-dependent bacteria, we mapped the KO detected in their genomes to the constructed SCFA generation, sulfur cycling, nitrogen cycling and fatty acid (FA) biosynthesis pathways, separately (Supplementary Figure 3 and Supplementary Table 6). The ANOVA analysis showed that pathway completeness, indicating metabolic capability, of hemicellulose degradation, pectin degradation, butyrate generation, and FA biosynthesis pathways were significantly different among the upregulated, downregulated, and unchanged populations of the community of microbes with Ni-dependent enzyme genes with the highest values in the upregulated population (Table 5).

**TABLE 5:** Comparison of completeness of pathways/sub-pathways among the populations of microbes with Ni-dependent enzyme genes.

(Sub)-Pathway	Pathway completeness^1^			*FDR*
	Upregulated population	Downregulated population	Unchanged population	
Starch degradation	5.22 ± 0.38	3.39 ± 0.60	4.32 ± 0.42	0.09
Glycolysis	15.02 ± 0.78	12.75 ± 0.67	13.58 ± 0.32	0.09
Cellulose degradation	2.79 ± 0.16	1.54 ± 0.33	2.28 ± 0.47	0.08
Hemicellulose degradation	15.44 ± 0.69	9.86 ± 1.41	13.45 ± 1.29	0.03
Pectin degradation	10.21 ± 0.78	6.76 ± 0.93	9.58 ± 0.96	0.04
Propionate generation	12.20 ± 0.63	9.26 ± 0.85	8.86 ± 1.39	0.10
Butyrate generation	7.10 ± 0.33	3.09 ± 0.73	2.31 ± 0.81	0.00
Acetate generation	4.72 ± 0.38	3.63 ± 0.51	3.88 ± 0.30	0.29
Formate generation	1.39 ± 0.08	0.94 ± 0.18	1.11 ± 0.23	0.28
Lactate metabolism	4.37 ± 0.46	2.60 ± 0.46	3.50 ± 0.77	0.13
Fatty acid biosynthesis	10.30 ± 0.75	6.86 ± 0.53	6.95 ± 1.17	0.02
Dissimilatory nitrate reduction	1.31 ± 0.33	0.48 ± 0.26	0.24 ± 0.24	0.07
Nitrogen fixation	0.48 ± 0.27	0.65 ± 0.21	0.46 ± 0.29	0.84
Assimilatory sulfate reduction	2.34 ± 0.67	1.75 ± 0.57	0.39 ± 0.39	0.11
Dissimilatory sulfate reduction	0.99 ± 0.40	0.90 ± 0.36	0.19 ± 0.19	0.28

*^1^Pathway completeness, pathway enzyme gene number/average completeness of bins.*

### Effects of Environmental Factors on the Structure of the Ruminal Microbiome and the Community of Microbes With Ni-Dependent Enzyme Genes

**FIGURE 2:**
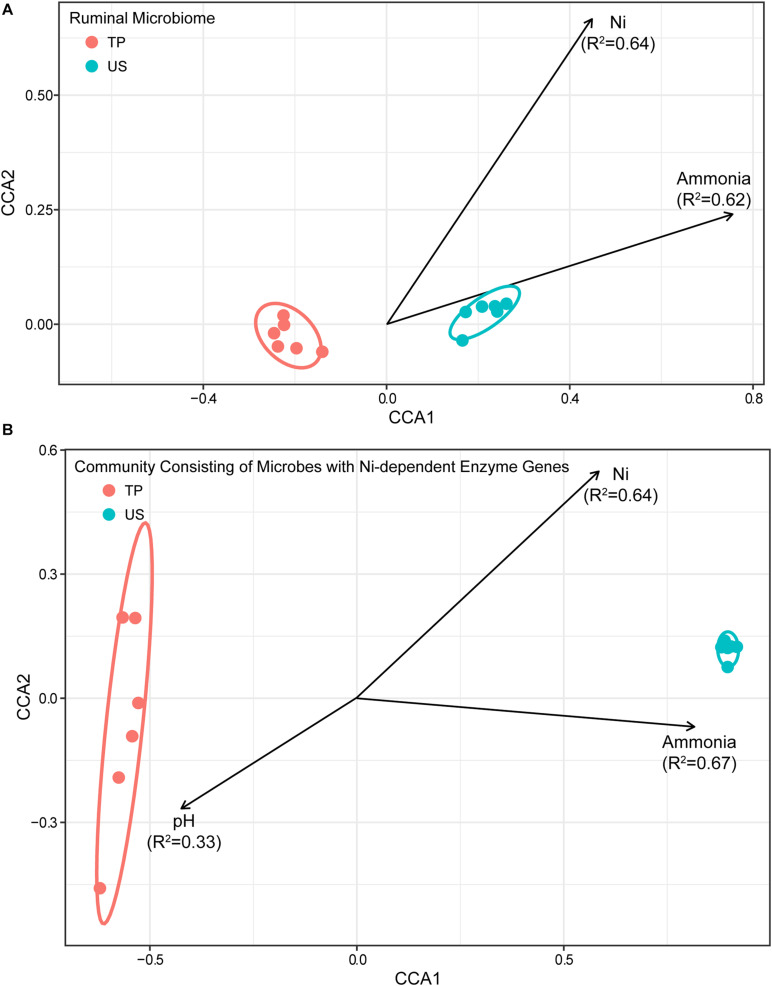
Constrained correspondence analysis plot depicting the relationship between rumen pH, Ni, and ammonia concentrations (environmental variables), and the structure of **(A)** the ruminal microbiome and **(B)** the community of microbes with Ni-dependent enzyme genes. Arrows represent the environmental variables that showed significant correspondence (FDR < 0.05) to the structure of the ruminal microbiome or community of microbes with Ni-dependent enzyme genes. Each point represents one rumen fluid sample. Arrow direction indicates the correspondence direction and arrow length indicates the importance/strength of the correspondence between the variable and the community structure.

To elucidate any relationships between environmental factors, including pH, Ni, ammonia, individual SCFA, L-lactate, and D-lactate concentrations on the structure of the ruminal microbiome, and that of the community with Ni-dependent enzyme genes, envfit function and CCA in the R vegan package were used to test their correlations. The results presented in Figure 2 showed that (1) the structure of the ruminal microbiome was significantly related to rumen Ni and ammonia concentrations, with squared correlation coefficients (*R*^2^) of 0.64 and 0.62, respectively; (2) the structure of the community of microbes with Ni-dependent enzyme genes was also related to ammonia and Ni concentrations with similar *R*^2^ values (0.67 and 0.64, respectively) but additionally correlated with rumen pH (*R*^2^ = 0.33). The relationship to pH was almost exactly opposite to the relationship with Ni concentration, indicating that concentrations of H^+^ and Ni were highly co-correlated with the structure of the community of microbes with Ni-dependent enzyme genes.

## Discussion

The present study was performed on young goats at an age where the rumen microbiome can be expected to be stably established (Han et al., 2015). We intended to investigate the compositional and functional changes of the ruminal microbiome by urea supplementation. Special focus was on the relevance of microbial changes for fermentation processes and, vice versa, a possible feed-back impact of fermentation-driven environmental variables (pH, SCFA, lactate, ammonia) on the ruminal microbiome. As urease is a Ni-dependent enzyme, the changes of the community consisting of microbes with Ni-dependent enzyme genes and its relation to Ni concentration and other environmental variables were of additional interest.

The US diet showed a slightly higher Ni content which, together with a higher dry matter intake (DMI) in the US group, might be one reason for the increase of ruminal Ni concentration after urea supplementation. Ni appears to be an essential trace element for ruminants. It is required in minute amounts and is essential for the maintenance of health, immunity, growth, production, and reproduction (Spears, 1984). Dietary Ni supplementation was shown to improve feed intake, feed efficiency and rumen urease activity in goats, calves, steers, and lambs (O’Dell et al., 1971; Anke et al., 1977; Spears et al., 1986; Oscar et al., 1987; Milne et al., 1990; Singh et al., 2019). The feed intake and growth effects during Ni supplementation were proposed to correlate with the activity of ruminal urease (Spears and Hatfield, 1980). Spears et al. (1977) found that a diet supplemented with 1% urea had no significant difference on the activity of ruminal urease when compared with the basal diet without urea supplementation. However, this study investigated neither the dietary Ni content nor the ruminal Ni concentration, having no hints on the impacts of Ni during urea supplementation. Our study suggested the importance of ruminal Ni availability for the ruminal urease activity during urea supplementation.

Out of the fermentation variables studied, all but propionate and butyrate concentrations were either changed or tended to be changed by the dietary treatment, indicating a functional reorganization of microbial fermentation in the rumen. With regard to nitrogen metabolism, urea supplementation increased ammonia concentration and urease activity, the last being also verified by increased abundance of *URE* sequences in the metagenome. The increase in rumen ammonia concentration was expected as it is a regular finding across almost all studies investigating the effect of dietary supplementation of urea in ruminants (Owens et al., 1980; Ludden et al., 2000; Taylor-Edwards et al., 2009). This effect of urea is also limiting the applicability of urea supplementation because high concentrations of ammonia are toxic to the animal and have to be avoided. Therefore, concurrent increases in urease activity are not desirable although they have been identified in many studies, including the present one. The lower the urease activity, the higher the benefits of urea application because ammonia release from urea should ideally be slow and continuous over the day (Patra and Aschenbach, 2018). At the metagenome level, the oversupply of ammonia-N by the US diet was additionally and conceivably associated with a (compensatory) reduction in the nitrogen metabolism pathway, as well as increases in ammonia incorporating pathways like alanine, aspartate and glutamate metabolism, lysine biosynthesis and pyrimidine metabolism.

Ureolysis provides net capture of two H^+^ per molecule urea and thus increases rumen pH (Aschenbach et al., 2011). By contrast, a decrease of rumen pH was observed in the present study. The latter suggests higher acid production rates, supported by a trend for higher concentrations of total SCFA and acetate in the rumen fluid. Higher SCFA concentrations plausibly fit the improved abilities of carbon fixation and FA biosynthesis, as well as the increased digestibility of NDF fiber (hemicellulose and pectin), the latter identified in the SCFA generation pathway of the ruminal microbiome. Results of urea supplementation on fiber digestibility have been variable in literature and may greatly depend on the type of NDF and additional factors. Some studies reported higher NDF digestibility after urea supplementation (Farmer et al., 2004; Ceconi et al., 2015), which was partially associated with increased rumen SCFA concentrations and increased feed efficiency (Ceconi et al., 2015). We here demonstrate that such stimulation of NDF digestibility can be explained by collective upregulation of enzyme clusters in the hemicellulose and pectin degradation pathways.

To elucidate the relationship between the fermentation products and the microbial community structure, we performed CCA. It is important to understand that the results of CCA are potentially bidirectional for all fermentation variables included. This means that significant correspondence between a fermentation variable and community structure may indicate that a certain level of fermentation variable may shape a certain community structure or that a certain community structure is producing a certain level of fermentation variable, or both. Interestingly, ammonia was the only fermentation variable that corresponded significantly to community structure of the ruminal microbiome. This underlines that the changes in the microbiome observed in the present study were primarily driven by the extra supply of ammonia (due to dietary urea supplementation) and not related to other (secondary) changes in fermentation like SCFA or lactate production. Vice versa, this also underlines the great resilience and redundancy of the ruminal microbiome for SCFA production, where similar fermentation patterns may result from rather different microbiome structures (Patra et al., 2019).

A unique finding of the present study was that microbial community structure was related to rumen Ni concentrations. As mentioned earlier, rumen Ni concentration varied between treatments, which was partly explainable by minor differences in dietary Ni content and increased feed intake of the US group. However, the difference in ruminal Ni concentration was larger (plus ∼40%) than its extrapolation from higher Ni intake (plus ∼13%) in the US group. The latter may indicate that some factors affecting rumen Ni concentrations were also changed by urea supplementation, such as ruminal fluid turnover rates, speed of Ni release from plant-based components during digestion, sequestration of Ni in ruminal microbiota and possibly also Ni absorption by the ruminal epithelium. Dietary Ni supplementation has been shown previously to increase urease activity in the rumen (Spears et al., 1977; Singh et al., 2019; Spears, 2019). By contrast, methane production, which requires the Ni-dependent cofactor F430 for coenzyme M reductase reaction, is mostly not influenced by dietary Ni supplementation (Oscar et al., 1987; Oscar and Spears, 1988). Virtually nothing is known about the regulation of other Ni-dependent enzymes or the microbes expressing those enzymes by Ni concentration in the rumen. The present study suggested that Ni might first affect the community of microbes with Ni-dependent enzyme genes and then extend its impacts on the whole ruminal microbiome. The structure of the community with Ni-dependent enzyme genes showed co-correlation with both rumen Ni and H^+^ concentrations. This comes as no surprise, because essentially all Ni-dependent catalysis is pH-dependent.

As indicated above, few reports have evaluated the distribution and abundance of Ni-dependent enzyme genes and microbes carrying those genes in the rumen, except for urease and ureolytic bacteria. In the present study, we detected and quantified five Ni-dependent enzyme genes using a metagenomic approach. Among them, the relative abundances of *ACS* and *URE* were increased, whereas the relative abundances of *GLO*, *HYD*, and *LAR* were decreased by urea supplementation. The functional importance of the decreased abundances of microbes with *GLO*, *HYD*, and *LAR* is not readily evident from the present study. However, it seems to be associated with improved fermentation as indicated by the trend for increased SCFA concentration. With regards to the increased abundance of *ACS*, most microbes carrying this enzyme gene have been shown to be acetogens that convert CO_2_ into acetate by using the reductive acetyl-CoA pathway (Ragsdale, 2007). Reductive acetogenesis catalyzed by the acetyl-CoA synthase is potentially a major pathway for improving the efficiency of fermentation by reducing methane emissions into the environment. It has been questioned previously that reductive acetogenesis plays any functional role in the rumen, which explains the high methane emissions from rumen compared to large intestine (Immig, 1996; Immig et al., 1996). Interestingly, a substantially higher amount of nitrogenous compounds in the caecum compared to the rumen has been suspected as one possibly important factor triggering reductive acetogenesis (Immig, 1996). Although *MCR* was not found in our metagenome sequences, which might relate to the low abundance of archaeal methanogens (0.16–0.43% of the total abundance of ruminal microbiome) and, especially, the scarce sequence information of archaeal genes in the available KO databases, the finding of increased *ACS* abundance after urea supplementation might have important implications as it indicates that reductive acetogenesis is potentially induced in the rumen by urea supplementation.

In total, 20 Ni-dependent bacteria have been identified as sources of Ni-dependent enzyme genes in the metagenome sequences of the present study. The results showed that the structure of this community, primarily depending on the numbers and the relative abundances of members, was different, whereas its diversity, primarily depending on the phylogenetic compositions, showed no significant difference between the treatments. Thus, our results suggested that urea supplementation affects the structure of the community of microbes with Ni-dependent enzyme genes but may have no significant impact on its functional stability. Among this community, *URE* has been found in *Treponema succinifaciens*, which is a known ureolytic bacterium in the rumen (Wozny et al., 1977). The presence of *ACS* has been allocated to three bacteria belonging to the order Clostridiales, which is consistent with previous studies (Mackie and Bryant, 1994). Consistent with the upregulation of the abundances of *URE* and *ACS*, the abundances of *Treponema succinifaciens* carrying *URE* tended to be upregulated and the abundances of bacteria carrying *ACS* were also upregulated by urea supplementation. A further important finding was that most of the microbes with Ni-dependent enzyme genes have more than one Ni-dependent enzyme gene encoded in their genomes. Ni-dependent enzymes have been shown to have metabolic interactions. For example, the [NiFe]-hydrogenase catalyzes the generation of H_2_, which is required by the acetogens in the generation of acetate. The glyoxalase I catalyzes the conversion of methylglyoxal into D-lactate and thus functions to detoxify this non-enzymatic byproduct of glycolysis (Suttisansanee et al., 2011). *LAR* has been found in the genomes of lactate-producing, acetogenic, sulfate-reducing, metal-reducing, fumarate-reducing, and butyrate-producing bacteria. Furthermore, co-cultures of two microbes with Ni-dependent enzyme genes have been shown to promote their metabolic abilities mutually (Kudo et al., 1987; Miron, 1991). Accordingly, we suggest that Ni-dependent enzymes establish metabolic connections for microbes with Ni-dependent enzyme genes, enabling them to alliance together, and therefore, help them to occupy the GI niche by sharing environmental benefits such as an increased local pH and synergetic metabolic abilities. On considering the abundances of Ni-dependent enzyme genes in the ruminal microbiome, only a small portion (∼5 to 45%) of the detected Ni-dependent enzyme sequences could be allocated to the 20 microbes with Ni-dependent enzyme genes in this study. Additionally, the pathway enrichment analysis of the microbes with Ni-dependent enzyme genes relied on the comparisons of gene abundance rather than the comparisons of gene expression and protein expression. These facts could explain the inconsistency between the upregulation of the butyrate production sub-pathway of these microbes and the missing effect on ruminal butyrate concentration. Hence, further studies are needed to obtain a more detailed picture of the composition and function of this community.

A major outcome of the present study is the elucidation of large functional overlaps between the effects of urea supplementation on the community of microbes with Ni-dependent enzyme genes with that on the ruminal microbiome. (1) Urea supplementation increases the abilities for the conversion of hemicellulose and pectin into SCFA for the microbes with Ni-dependent enzyme genes with concurrent increases in the abilities of hemicellulose and pectin degradation for the ruminal microbiome. (2) Urea supplementation increases the abilities of FA biosynthesis for the microbes with Ni-dependent enzyme genes and the ruminal microbiome. (3) Urea supplementation decreases the abundances of the important starch degraders *Selenomonas ruminantium* and *Succiniclasticum ruminis* (Marounek and Bartos, 1986; Han and Chen, 1988) within the community of microbes with Ni-dependent enzyme genes and decreases the ability of starch degradation for the ruminal microbiome. (4) Urea supplementation decreases the abundance of *HYD* with its essential roles in sulfate/sulfide and nitrate/nitrite reduction (Mulrooney and Hausinger, 2003) within the community of microbes with Ni-dependent enzyme genes and also decreases the abilities of sulfur metabolism and nitrogen metabolism of the ruminal microbiome. (5) Urea supplementation increases the abundance of *ACS* with an ability to fix carbon in the Ni-dependent community with parallel increases in the ability of carbon fixation in the ruminal microbiome. Finally, the identification of rumen Ni and ammonia concentrations as the common environmental determinants, not only for the microbes with Ni-dependent enzyme genes, but also for the whole ruminal microbiome, is highly suggestive of a causative relationship. The last-mentioned can be taken as an indication that the compositional changes of the community of microbes with Ni-dependent enzyme genes may be a key event driving the compositional and functional changes of the ruminal microbiome.

In the present study, we selected bins with specific gene markers to investigate the types and function of specific microbes within the community. So far, our knowledge on the kinds of ruminal microbes taking roles in the specific metabolic pathways, such as lactate metabolism and urea hydrolysis, were highly dependent on culture-based methods. However, these *in vitro* results may not adequately reflect the real metabolic processes occurring in the rumen. Furthermore, some important microbes involved in these metabolic processes cannot be cultured yet. We suggest that the selection of bins with specific gene markers is a promising way to identify these specific microbes from the metagenome sequencing results. However, it should be noted that the accuracy of this method is highly dependent on the maturity of available binning and taxonomy annotation methods, as well as the comprehensiveness of available genome and gene databases, all of which are currently in a developing stage. The latter explains that we were able to detect only 20 microbes with Ni-dependent enzyme genes in our data set and that the compositions of the archaeal community of the present study is not consistent with that indicated by conventional 16S rRNA gene sequencing where genus *Methanobrevibacter* is commonly most abundant within the rumen archaeal community (Whitford et al., 2001; Franzolin et al., 2012; Paul et al., 2017). Nonetheless, the applied approach of metabolic profiling based on metagenome data has high potential for studying the *in vivo* function of ruminal microbes and is, as such, a powerful supplementation to culture-based methods.

In conclusion, the present study indicates that urea supplementation affects the composition and function of the ruminal microbiome and these effects are largely mirrored by effects on the community of microbes with Ni-dependent enzyme genes. The observed changes in the frequency of genes support improved capacities for hemicellulose and pectin-type fiber digestion, improved capacities for FA biosynthesis and carbon fixation, decreased capacity for starch degradation and changes in sulfur and nitrogen metabolism pathways. Although these functional changes were deducted from gene frequency data rather than gene expression or enzyme activity data, they largely fit the alterations in fermentation pattern observed in the present and previous studies. The data thus extends our view on the possible mechanisms underlying the changes in fermentation pattern during urea supplementation. Given the fact that the changes in microbiome structure after urea supplementation were not only related to rumen ammonia concentration but also to rumen Ni concentration, it may be proposed that the amount of dietary Ni intake and microbial Ni retention in the rumen may shape the effects of urea supplementation on rumen fermentation. The latter suggestions should be explored in future studies.

## Data Availability Statement

The datasets presented in this study can be found in online repositories. The names of the repository/repositories and accession number(s) can be found below: https://www.ncbi.nlm.nih.gov/, BioProject PRJNA622657.

## Ethics Statement

The animal study was reviewed and approved by the Animal Care and Use Committee of Nanjing Agricultural University. Written informed consent was obtained from the owners for the participation of their animals in this study.

## Author Contributions

HS and ZL conceived and designed the experiments. ZL and LK performed the animal feeding experiments, DNA extraction, rumen fermentation indices determination, and metagenome sequencing libraries preparation. HS and ZX performed the bioinformatic analyses. HS, ZL, and JA interpreted the results and drafted the manuscript. All authors read and approved the final manuscript.

## Conflict of Interest

The authors declare that the research was conducted in the absence of any commercial or financial relationships that could be construed as a potential conflict of interest.

## Footnotes

1 http://broadinstitute.github.io/picard/

2 http://www.ncbi.nlm.nih.gov/RefSeq/

3 https://www.uniprot.org/

## Supplementary Material

Supplementary Figure 1 Non-metric multidimensional scaling (NMDS) plot visualizing the effects of urea supplementation on the compositions of **(A)** the ruminal microbiome and **(B)** the community of microbes with Ni-dependent enzyme genes. Each dot represents one rumen fluid sample. NMDS analysis indicated a significant difference between the two groups (FDR < 0.05).

Supplementary Figure 2 Changes of KEGG metabolism pathways (excluding carbohydrate metabolism pathways) of the ruminal microbiome attributable to urea supplementation. The size of the dots represents the ratio of significantly upregulated/downregulated gene numbers on the specific pathway to that of the whole community. The significance level is color-coded.

Supplementary Figure 3 Schematic representation of sulfur cycling, nitrogen cycling, and fatty acid biosynthesis pathways of the microbes with Ni-dependent enzyme genes.

Supplementary Table 1 Effects of urea supplementation on the relative abundances of archaea and bacteria in the ruminal microbiome.

Supplementary Table 2 Effects of urea supplementation on the diversity of ruminal microbiome and the community of microbes with Ni-dependent enzyme genes.

Supplementary Table 3 Effects of urea supplementation on the relative abundances of KO terms in the ruminal microbiome.

Supplementary Table 4 Effects of urea supplementation on the relative abundances of pathway enzyme genes and KO terms of the constructed SCFA generation pathway for the ruminal microbiome.

Supplementary Table 5 Relative abundances and taxonomic annotation of 57 clusters (bins) with Ni-dependent enzyme genes that showed >60% completeness, <10% contamination, and <10% strain heterogeneity.

Supplementary Table 6 Pathway mapping results of KO terms of 20 microbes with Ni-dependent enzyme genes.
