# Supplementary material for: Functional Changes of the Community of Microbes With Ni-Dependent Enzyme Genes Accompany Adaptation of the Ruminal Microbiome to Urea-Supplemented Diets
Source: Front Microbiol. 2020 Dec 22;11:596681. doi: 10.3389/fmicb.2020.596681 (PMC7782429; doi:10.3389/fmicb.2020.596681)
Supplement: Supplementary Figure 1 — Non-metric multidimensional scaling (NMDS) plot visualizing the effects of urea supplementation on the compositions of (A) the ruminal microbiome and (B) the community of microbes with Ni-dependent enzyme genes. Each dot represents one rumen fluid sample. NMDS analysis indicated a significant difference between the two groups (FDR < 0.05). [file Data_Sheet_1.PDF]

Supplementary Material

1 Supplementary Figures

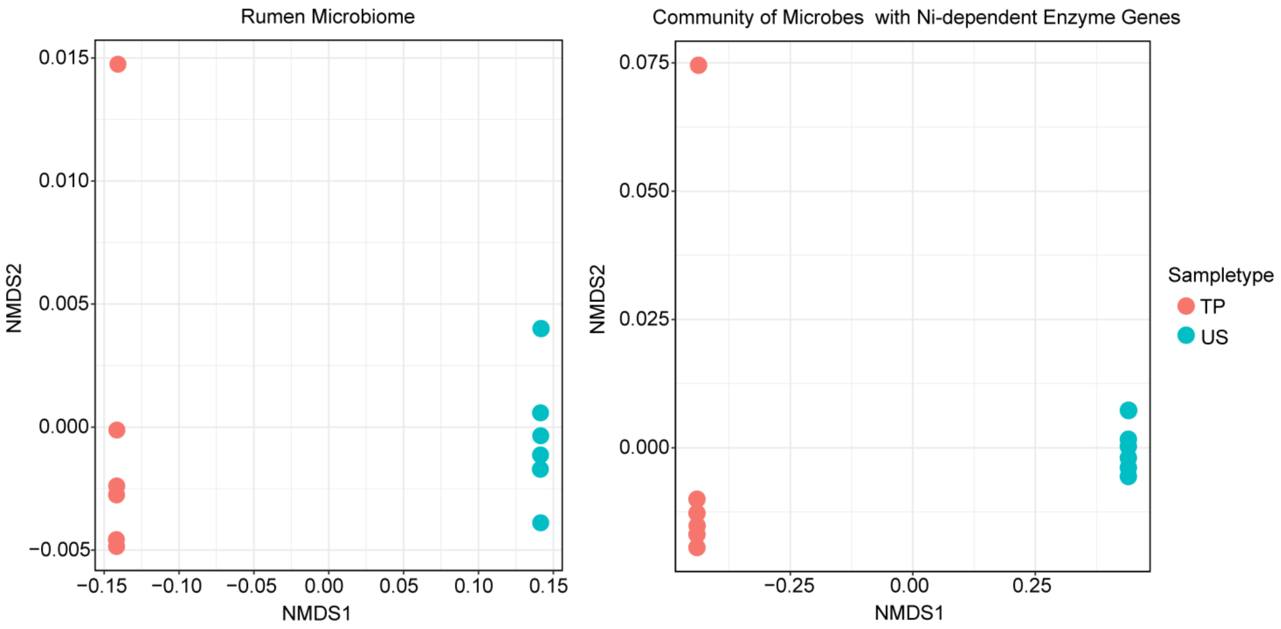

Supplementary Figure 1. Non-metric multidimensional scaling (NMDS) plot visualizing the effects of urea supplementation on the compositions of A) the ruminal microbiome and B) the community of microbes with Ni-dependent enzyme genes. Each dot represents one rumen fluid sample. NMDS analysis indicated a significant difference between the two groups (FDR < 0.05).

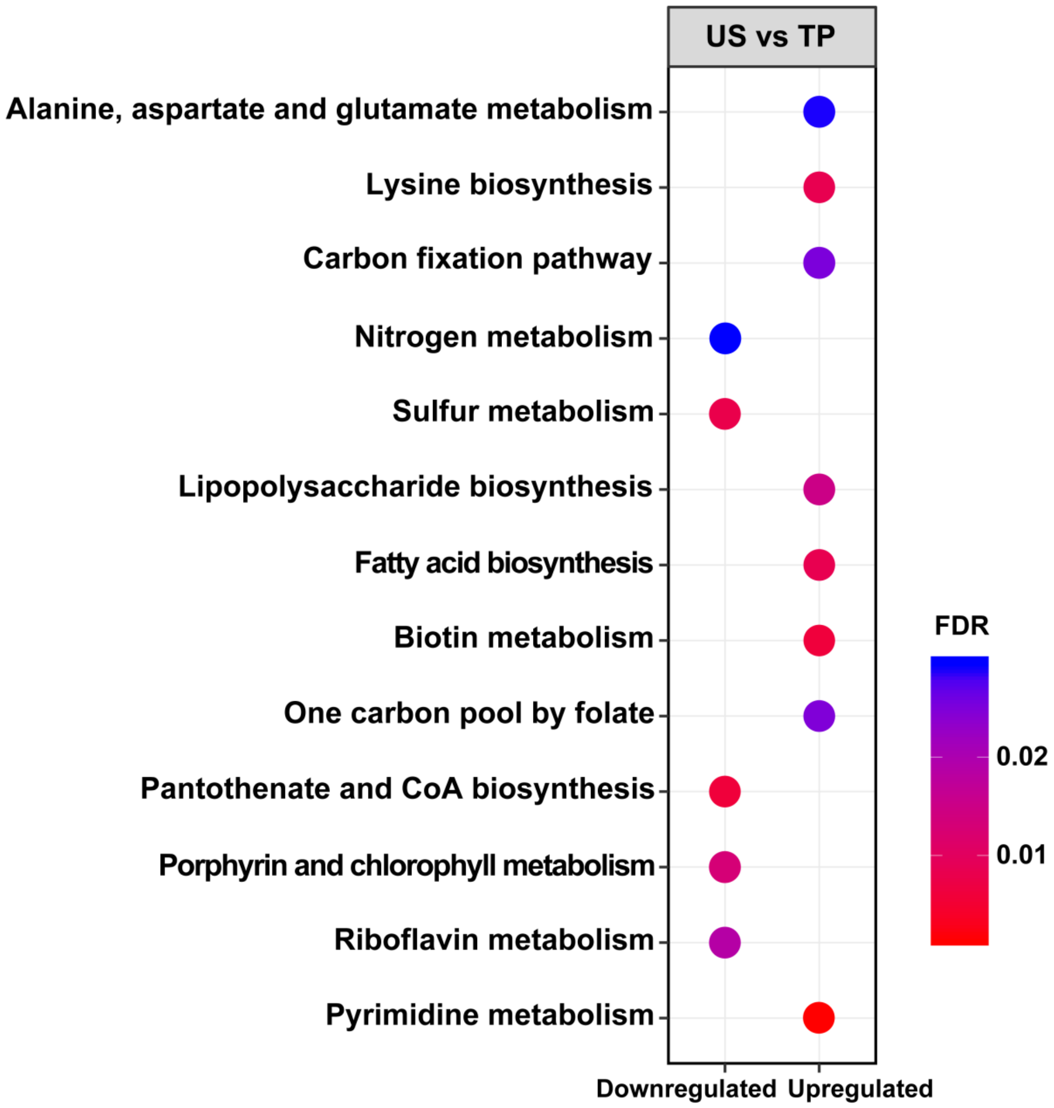

Supplementary Figure 2. Changes of KEGG metabolism pathways (excluding carbohydrate metabolism pathways) of the ruminal microbiome attributable to urea supplementation. The significance level is color-coded.

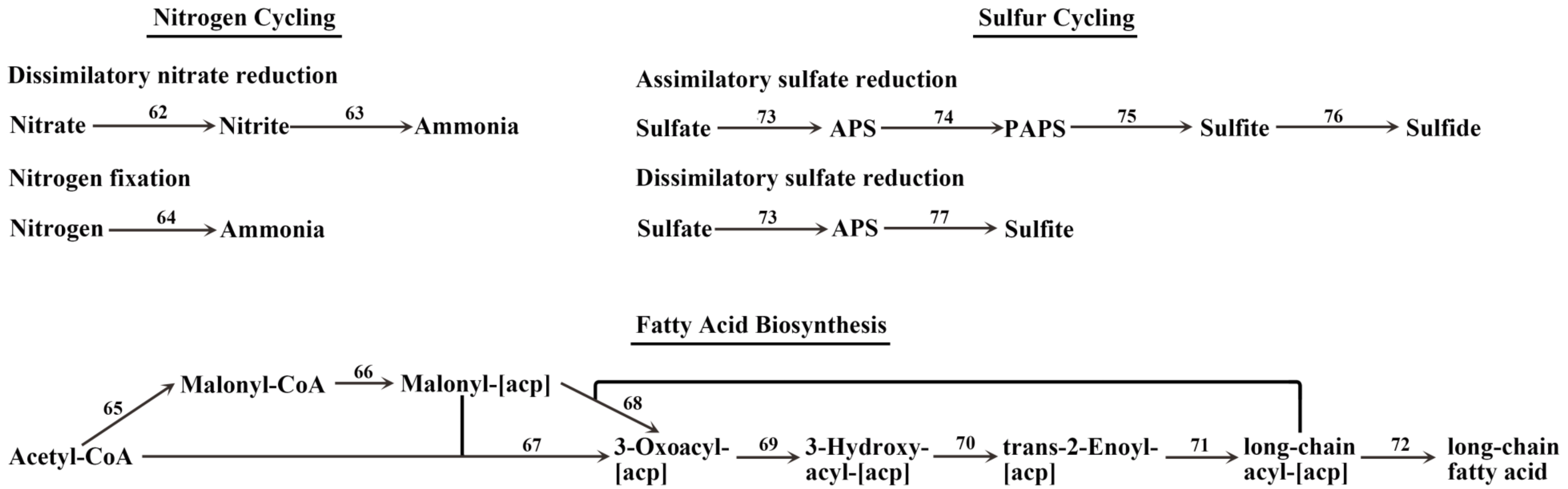

Supplementary Figure 3. Schematic representation of sulfur cycling, nitrogen cycling, and fatty acid biosynthesis pathways of the microbes with Ni-dependent enzyme genes.
